# Modulation of *Candida albicans* virulence in *in vitro* biofilms by oral bacteria

**DOI:** 10.1111/lam.13145

**Published:** 2019-03-21

**Authors:** D.J. Morse, M.J. Wilson, X. Wei, D.J. Bradshaw, M.A.O. Lewis, D.W. Williams

**Affiliations:** ^1^ School of Biosciences Cardiff University Cardiff UK; ^2^ School of Dentistry Cardiff University Cardiff UK; ^3^ GlaxoSmithKline Consumer Healthcare Weybridge UK

**Keywords:** biofilms, diseases, fungi, gene expression, virulence

## Abstract

*Candida*‐associated denture stomatitis presents as erythema of the palatal mucosa and is caused by biofilms containing the fungus *Candida albicans* that co‐reside with oral bacteria on the denture‐fitting surface. This study aimed to assess the effect of several frequently encountered oral bacteria on the expression of *C. albicans* virulence factors in *in vitro* polymicrobial biofilms. Biofilms containing *C. albicans* and selected bacterial species were grown on denture acrylic, and analysed by microscopy and by qPCR for expression of putative virulence genes. *Candida albicans*‐only biofilms showed limited hyphal production. Hyphal development was significantly (*P* < 0·001) increased when biofilms also contained four species of oral bacteria (*Streptococcus sanguinis*,* Streptococcus gordonii*,* Actinomyces odontolyticus* and *Actinomyces viscosus*), as was the expression of virulence genes (*P* < 0·05). Importantly, inclusion of *Porphyromonas gingivalis* in the biofilm consortium resulted in significant (*P* < 0·05) inhibition of virulence gene expression and production of hyphae. The *in vitro* expression of *C. albicans* virulence factors was modulated in polymicrobial biofilms. The complexity of this modulation was highlighted by the reversal of effects following introduction of a single bacterial species into a biofilm community.

**Significance and Impact of the Study:**

The impact of individual bacterial species on *Candida albicans* virulence highlights both the complexity of predicting infection mediated by polymicrobial communities and the potential for management through pro‐ or prebiotic therapy. The possibility to selectively modulate microbial virulence by addition of, or treatment with pro‐ or prebiotics avoids the use of conventional antimicrobial compounds, thus reducing the contribution to potential drug resistance. Understanding which bacterial species modulate virulence, and the mechanisms by which this occurs, particularly in biofilms, provides excellent foundations for further research questions, and the potential for novel clinical interventions.

## Graphical abstract

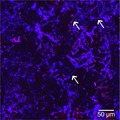

## Introduction

The fungal genus *Candida* consists of a number of important species (Haynes 2001; Ganguly and Mitchell 2011; Patil *et al*. 2015), some of which are implicated in localized and systemic human infection (Ganguly and Mitchell 2011; Patil *et al*. 2015). Collectively, these infections are known as candidoses (Scully *et al*. 1994; Williams and Lewis 2011), and tend to occur in people with one or more predisposing factors, including a weakened immune system, diabetes, human immunodeficiency virus (HIV)/acquired immune deficiency syndrome (AIDS) or altered microbiota as a result of antibiotic or steroid use (Williams and Lewis 2011; Patil *et al*. 2015). In addition, controllable predisposing factors include prolonged denture‐wearing and while sleeping, tobacco use and an ill‐fitting denture (Shulman *et al*. 2005; Rogers *et al*. 2013).

In the oral cavity, *Candida*‐associated infections are termed oral candidoses, of which there are four primary clinical presentations: chronic and acute erythematous, pseudomembranous and chronic hyperplastic candidosis (Patil *et al*. 2015). Chronic erythematous candidosis, also referred to as denture‐associated stomatitis (DS) (Barnabé *et al*. 2004; Salerno *et al*. 2011), presents symptomatically as inflammation of the palatal mucosa, and when diagnosed, is categorized into one of the four severities: Type 0, no erythema; Type 1, localized, pinpoint erythema; Type 2, more diffuse erythema, including part or all of the palate; and Type 3, severe erythema/papillary hyperplasia, often including the alveolar ridge (Samaranayake *et al*. 2002; Coco *et al*. 2008; Dar‐Odeh *et al*. 2012). While symptoms of itching, burning or generalized oral discomfort may be mild, their chronic nature necessitates management and treatment.

*Candida*‐associated denture stomatitis occurs due to the presence of polymicrobial biofilms containing *Candida* on the fitting‐surface of the denture. These biofilms are in prolonged contact with the palate (Coco *et al*. 2008; Gendreau and Loewy 2011) and this leads to a host inflammatory response and the characteristic red colouration and inflammation of the palate (Rogers *et al*. 2013).

In laboratory studies, we have previously shown that co‐culturing *C. albicans* with a range of oral bacteria in biofilms on denture acrylic materials resulted in enhancement of *C. albicans* virulence (Cavalcanti *et al*. 2015; Morse *et al*. 2018). This enhanced virulence led to increased tissue damage and invasion in tissue models. However, with the oral bacterial microbiota consisting of upwards of 700 species (Aas *et al*. 2005; Chen *et al*. 2010; Dewhirst *et al*. 2010), including additional bacterial species to make the biofilm model more clinically representative is of great interest. In the present study, the inclusion of *Porphyromonas gingivalis* in the biofilm model was undertaken to assess its effect on subsequent markers of *C. albicans* virulence.

## Results and discussion

**Figure 1:**
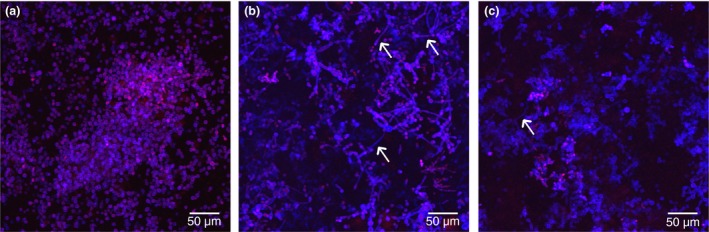
Typical confocal microscope images of biofilms containing (a) *Candida albicans*‐only, (b) mixed‐species inoculum (*C. albicans* plus four oral bacterial species), and (c) mixed‐species inoculum plus *Porphyromonas gingivalis*. Biofilms stained with calcofluor white and propidium iodide. The white arrows indicate *C. albicans* hyphae. [Colour figure can be viewed at http://wileyonlinelibrary.com]

**Figure 2:**
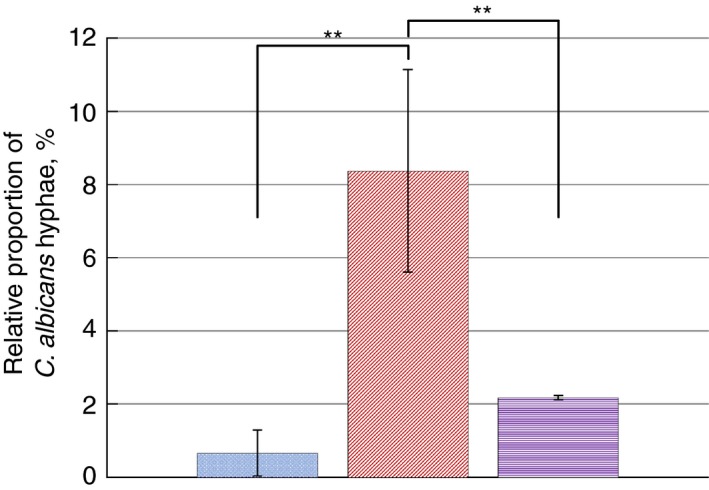
Comparison of average number of *Candida albicans* hyphae, expressed as a percentage of the number of total cells (including yeast and hyphae) between *C. albicans*‐only biofilms (left‐most, blue dotted‐texture bar), mixed‐species biofilms (middle, red diagonal‐texture bar) and mixed‐species plus *Porphyromonas gingivalis* biofilms (right‐most, purple horizontal‐texture bar). A significant increase in the number of observed hyphae was evident in mixed‐species biofilms relative to *C. albicans*‐only and mixed‐species plus *P. gingivalis* biofilms. (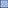) *C. albicans* only, (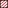) mixed‐species and (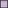) mixed‐species + *P. gingivalis*. [Colour figure can be viewed at http://wileyonlinelibrary.com]

The formation of hyphae is considered an important virulence factor of *C. albicans*. Culture of *C. albicans* biofilms without other microorganisms resulted in limited hyphal development (Table 1), which were at a basal level in the biofilm structure (Figs 1a and 2). However, biofilms containing *C. albicans* with mixed‐species of oral bacteria (consisting of *Streptococcus sanguinis, Streptococcus gordonii, Actinomyces viscosus* and *Actinomyces odontolyticus*) resulted in over a 12‐fold increase in the proportion of hyphae relative to yeast cells (Figs 1b and 2), which was statistically significant (*P* < 0·001). Including *P. gingivalis* in the *C. albicans* mixed‐species biofilm consortium, led to a substantial reduction in the previously observed increase in hyphal formation (Figs 1c and 2). This significantly (*P* < 0·001) lower level of *C. albicans* hyphae relative to the mixed‐species biofilms was similar to that of the *C. albicans*‐only biofilms, with only a slight but not‐statistically significant increase evident (*P* = 0·42).

**Table 1:** Relative proportion of hyphae in biofilms containing *Candida albicans*‐only, mixed‐species (*C. albicans* plus four oral bacterial species), and mixed‐species plus *Porphyromonas gingivalis*

Sample	Relative proportion of hyphae (%)		
	*C. albicans* only	Mixed‐species	Mixed‐species plus *P. gingivalis*
1	0·82	3·88	2·11
2	0·00	8·29	2·23
3	0·21	10·75	2·13
4	1·62	10·57	2·22
Mean (SD)	0·66 (0·62)	8·37 (2·77)	2·18 (0·06)

In addition to hyphal formation evident by microscopy, qPCR was employed to quantify expression of putative virulence genes associated with biofilm formation and production of hydrolytic enzymes. The relative gene expression was compared with the housekeeping gene *ACT1*, normalised to *C. albicans*‐only biofilms. The *ACT1* gene is involved in production of actin for cytoskeletal functions, and the consistent expression of the gene is widely used as a suitable housekeeping gene for qPCR analyses (Alves *et al*. 2014; Komalapriya *et al*. 2015; Alonso *et al*. 2018). No significant differences were observed in the number of viable *C. albicans* cells in either *C. albicans*‐only or mixed‐species biofilms.

**Figure 3:**
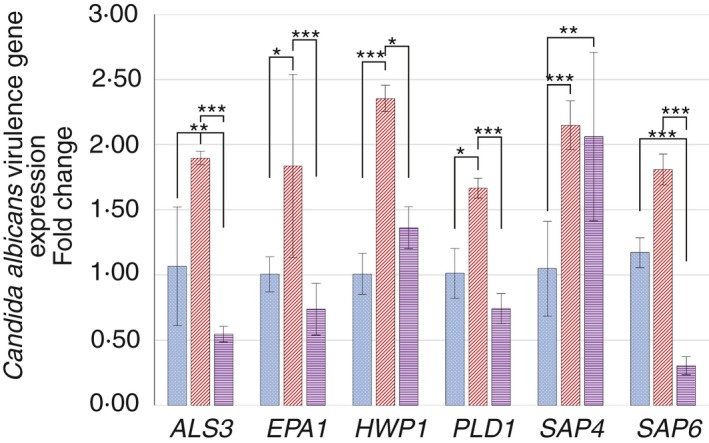
Relative expression of *Candida albicans* virulence genes, comparing *C. albicans*‐only biofilms (left‐most, blue dotted‐texture bars), mixed‐species biofilms (middle, red diagonal‐texture bars) and mixed‐species plus *Porphyromonas gingivalis* biofilms (right‐most, purple horizontal‐texture bars). A significant increase in the expression of all evaluated virulence genes was observed in mixed‐species biofilms compared with *C. albicans*‐only biofilms. An increase of virulence gene expression was not typically observed in mixed‐species plus *P. gingivalis* biofilms compared with *C. albicans*‐only biofilms, with the exception of *SAP4*. *ALS3*,*HWP1* and *EPA1* genes express adhesins for attachment to various surfaces and biofilm formation. *PLD1*,*SAP4* and *SAP6* genes are involved in production of secreted hydrolytic enzymes involved in nutrient acquisition, hyphal formation (also Als3, Hwp1), and tissue invasion and damage. (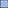) *C. albicans* only, (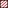) mixed‐species and (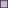) mixed‐species + *P. gingivalis*. [Colour figure can be viewed at http://wileyonlinelibrary.com]

Changes in the relative expression of a number of *C. albicans* virulence genes, as presented in Fig. 3 and Table 2, was evident in mixed‐species (middle, red diagonal‐textured bars) and mixed‐species plus *P. gingivalis* biofilms (right‐most, purple horizontal‐textured bars) compared with *C. albicans*‐only biofilms (left‐most, blue dotted‐texture bars).

**Table 2:** Changes in *Candida albicans* gene expression of mixed‐species and mixed‐species plus *Porphyromonas gingivalis* biofilms relative to expression from *C. albicans*‐only biofilms

*C. albicans* virulence gene	Change in expression, relative to *C. albicans*‐only biofilms			
	Mixed‐species	Sig.	Mixed‐species plus *P. gingivalis*	Sig.
ALS3	Increase	*P* < 0·01	Decrease	*P* < 0·01
EPA1	Increase	*P* < 0·01	No change	*P* > 0·05
HWP1	Increase	*P* < 0·001	No change	*P* > 0·05
PLD1	Increase	*P* < 0·05	No change	*P* > 0·05
SAP4	Increase	*P* < 0·001	Increase	*P* < 0·01
SAP6	Increase	*P* < 0·05	Decrease	*P* < 0·001

Compared with *C. albicans*‐only biofilm controls, mixed‐species biofilms had a consistently higher expression of all measured *C. albicans* virulence genes: agglutinin‐like sequence (*ALS3*,* P* < 0·01), epithelial adhesin (*EPA1*,* P* < 0·01); hyphal wall protein 1 (*HWP1*,* P* < 0·001), phospholipase D 1 (*PLD1*,* P* < 0·001), and secreted aspartyl proteinases 4 and 6 (*SAP4*,* P* < 0·001; *SAP6, P* < 0·05).

Mixed‐species biofilms including *P. gingivalis* resulted in a different pattern of *C. albicans* gene expression compared with *C. albicans*‐only biofilms. In these cases, only one virulence gene, *SAP4*, was significantly (*P* < 0·01) elevated, and this was at a similar relative expression as observed in the mixed‐species biofilms. Despite an increase in *SAP4* gene expression, the level of expression of other genes involved in biofilm formation and hyphal transition was lower, including *ALS3* (*P* < 0·01) and *SAP6* (*P* < 0·001). All other virulence genes including *EPA1*,* HWP1* or *PLD1* showed no significant (*P* > 0·05) changes compared with *C. albicans*‐only biofilms.

The pattern of increased *C. albicans* gene expression, particularly those involved in hyphal formation (*ALS3*,* HWP1*,* SAP4*), correlated with visible *Candida* morphology in *C. albicans*‐only and mixed‐species biofilms (Fig. 1). Furthermore, the reduced expression of these genes when *P. gingivalis* was included in mixed‐species biofilms was associated with lower numbers of visible *C. albicans* hyphae (Fig. 1c).

There is a need to better understand the interactions between microorganisms within a biofilm, how such interactions may influence the local biofilm environment and the resulting effects on pathogenic outcomes. In the case of the oral microbiome, and particularly interactions between bacteria and fungi, the influence of bacterial species in modulating the virulence capacity of *C. albicans* is of great interest. This has wider implications in terms of the clinical application of pre/probiotics to reduce the burden of specific bacterial species which, for example, may be modulating virulence disproportionately to its relative abundance.

The results of this study showed that incorporation of a single bacterial species into a polymicrobial inoculum consisting of *C. albicans* and four oral bacterial species resulted in a different virulence profile for *C. albicans*. The selected oral bacteria used in this study are frequently recovered from the oral cavity and associated with denture‐residing biofilms (Campos *et al*. 2008; Chen *et al*. 2010; Jenkinson 2011). The previously observed significant enhancement of *C. albicans* virulence gene expression related to biofilm formation and production of hydrolytic enzymes involved in increasing tissue damage and invasion (Naglik *et al*. 2003, 2004; Ganguly and Mitchell 2011; Morse *et al*. 2018) was not evident in biofilms including *P. gingivalis*. Both contact dependent and secreted/soluble factor interactions between *S. gordonii* and *P. gingivalis* have previously been observed (Avila *et al*. 2009), and both bacterial species have also been implicated in interactions with *C. albicans* and subsequent virulence factor expression (Thein *et al*. 2006; Nobbs *et al*. 2010; Bamford *et al*. 2015; Jack *et al*. 2015). This suggests that even within a complex polymicrobial biofilm, interactions between pairs of microbial species may play a part in the overall behaviour, or contribute to environmental changes that, for example, encourages virulence.

The mechanism of both enhancement of virulence by the bacteria contained within the mixed‐species biofilms, and its reversal when further incorporating an additional bacterial species is of much interest. It is possible that the mechanism(s) involved could require physical interaction or be mediated via secreted/soluble factors (Bandara *et al*. 2013). The mixed‐species bacteria contained streptococci, which are acidogenic and aciduric. Acidic conditions also tend to be favoured by *C. albicans*, while hyphal induction is restricted (Sudbery 2011). This indicates that additional mechanisms are likely to be involved, such as specific ligand‐receptor interactions, and/or soluble bacterial products.

Interactions between specific bacteria and *C. albicans* have been reported to induce expression of virulence factors (Silverman *et al*. 2010; Jack *et al*. 2015). However, these models are either limited to planktonic phase or restricted in that they do not represent the complexity of the oral microbiota, which is difficult to achieve *in vitro* due to the many varying culture conditions necessary to support the growth of all types of microorganisms involved. Indeed, our biofilm model is clearly still limited in terms of achieving the true diversity that exists *in vivo*, but does provide an advancement towards a more complex community with the incorporation of multiple bacterial species of different genera, and the fungus *C. albicans*. This work also reinforces the need to develop more complex models, incorporating additional microbial components, as the effect of even one added species into a mixed community can have, as observed in this study, substantial influence on microbial behaviour in the community.

The impact of including an additional single bacterial species in this biofilm model, and the subsequent changes in *C. albicans* behaviour also highlights the possibility of selectively modulating the behaviour of microorganisms in biofilms and/or infections in general. In the case of *P*. *gingivalis*, secreted molecules such as gingipains play a significant role in modulation of bacterial virulence (Genco *et al*. 1999; O'Brien‐Simpson *et al*. 2003; Potempa *et al*. 2003), although the mechanism by which this occurs is not currently known. *Porphyromonas gingivalis* is itself a known pathogen, and not necessarily the optimal solution for modulation of virulence, but this relationship between *P. gingivalis* and the biofilm community, with specific consideration of *C. albicans* virulence within these biofilms, emphasizes the potential implications of preferentially modifying the biofilm community behaviour away from virulence, but towards commensalism.

In summary, the contribution of members of the oral microbiota is an important consideration when modelling disease or infection caused by a single species such as *C. albicans*. Inclusion or exclusion of specific species can have profound effects on the behaviour of other micro‐organisms, many of which adapt their behaviour based on the presence of other members within the biofilm community.

## Materials and methods

### Microorganisms and culture conditions

Microorganisms used in the study were cultured and maintained under the following conditions.

*Candida albicans* ATCC 90028 was maintained on Sabouraud Dextrose Agar (SDA; LabM, Lancashire, UK), and cultured in yeast nitrogen base (YNB; BD Difco, Oxford, UK) supplemented with 100 mmol L^−1^ glucose (Fisher Scientific, Loughborough, UK) overnight under aerobic conditions and at 37°C. The oral bacterial species used were *S. sanguinis* NCTC 7863, *S. gordonii* ATCC 10558, *A. viscosus* ATCC 1598, *A. odontolyticus* NCTC 9935 and *P. gingivalis* NCTC 11834. Streptococci were cultured aerobically on blood agar (BA; Blood Agar Base, LabM, Heywood, UK) supplemented with 5% (v/v) defibrinated horse blood (TCS Biosciences, Buckingham, UK) at 37°C. For liquid culture, streptococci were cultured in Brain Heart Infusion (BHI) broth (LabM) under aerobic conditions at 37°C. *Actinomyces* species and *P. gingivalis* were maintained on fastidious anaerobe agar (LabM) supplemented with 5% (v/v) defibrinated horse blood (TCS Biosciences), and for liquid culture, were cultured in BHI supplemented with 50 *μ*g hemin per ml (Sigma, Gillingham, UK) and 10 *μ*g vitamin K per ml, under anaerobic conditions at 37°C.

### Biofilm development

Using a spectrophotometer (DiluPhotometer™, Implen, Westlake Village, CA), *C*. *albicans* and bacteria were adjusted to an OD_600 nm_ of 1·00 (±0·05) and 0·09 (±0·01), respectively.

Biofilms were cultured as previously described previously (Cavalcanti *et al*. 2015; Morse *et al*. 2018). Briefly, sterile poly‐(methyl methacrylate) (PMMA) discs (*c*. 10 mm diameter and 2 mm thickness) were preconditioned overnight with artificial saliva (containing 2·5 g porcine stomach mucin per litre (Sigma), 0·35 g sodium chloride per litre, 0·2 g potassium chloride per litre, 0·2 g calcium chloride dehydrate per litre, 2 g yeast extract per litre, 1 g Lab‐Lemco powder per litre (Sigma), 5 g proteose peptone per litre (Sigma) and 1·25 ml 40 % (w/v) urea solution per litre in sterile distilled water). The discs were then aseptically placed into sterile 24‐well plates, and 100 *μ*l of each standardized microbial culture was added to the surface of the discs. Biofilm preparations comprised of *C. albicans*‐only, mixed‐species (*C. albicans* plus *S. sanguinis, S. gordonii, A. viscosus, A. odontolyticus*), and mixed‐species with *P. gingivalis*. Sterile Dulbecco's Modified Eagle Medium (DMEM) (supplemented with 10% (v/v) foetal bovine serum and 50 mmol l^−1^ L‐glutamine per litre) was added to a final volume of 2 ml in each well. Culture medium was allowed to acclimatise in 5% CO_2_/95% air incubator for 1 h prior to use to ensure true and consistent representation of CO_2_ gaseous conditions. Microorganisms were cultured in 5% CO_2_/95% air and allowed to adhere to the discs for 90 min under agitation. Following incubation, the culture medium and non‐adherent cells were removed, and 2 ml of fresh culture medium was added. The discs were incubated for a further 72 h with a daily change of culture medium, then removed for respective analyses.

### Confocal microscopy to quantify *C. albicans* hyphae

Biofilms cultured on PMMA discs were fixed with 4% (v/v) formal saline. Thirty *μ*l of propidium iodide (PI) (Live/Dead™ *Bac*Light bacterial viability kit (Thermo Fisher Scientific, Paisley, UK)) and calcofluor white (diluted 1 : 100 with sterile distilled water) (CW) (Sigma) were applied to the surface of the PMMA discs and incubated at room temperature in the dark for 30 min to bind to nucleic acid (PI) or *C. albicans* cell wall (CW). Confocal laser scanning microscopy (CLSM) was then performed using a Leica TCS SP2 AOBS spectral confocal microscope (Leica Microsystems GmbH, Wetzlar, Germany).

Representative images of both dye channels were obtained from a minimum of four fields of view. Images were analysed by ImageJ 1.46r (Wayne Easband, National Institute of Health, Bethesda, MD). The images were adjusted by threshold intensity and the ‘count particles’ function was used to distinguish yeast cells from elongated hyphae, where both counted (hyphae counted manually, and yeast cells counted automatically). The proportion of hyphae relative to the number of yeast cells was calculated and analysed by one‐way analysis of variance, with Tukey's multiple comparisons test, at 95% confidence.

### *Candida albicans* virulence gene expression

Total RNA was extracted from the biofilms as previously described (Cavalcanti *et al*. 2015; Morse *et al*. 2018). Briefly, biofilms were suspended in lysis buffer (Qiagen, Crawley, UK) containing 1% (v/v) *β*‐mercaptoethanol. The biofilms were disrupted mechanically by high‐speed homogenization using glass beads in a Mini‐Bead‐Beater‐8 (Stratech Scientific, Soham, UK) for 1 min. Separation of nucleic acid from cell debris and proteins was achieved with phenol : chloroform : isoamyl alcohol (25 : 24 : 1) (Sigma), and total RNA was recovered after DNase I (Qiagen) treatment, and purified using the RNeasy Mini Kit (Qiagen) according to the manufacturer's instructions.

Reverse transcription for cDNA synthesis was performed using a Precision nanoScript2 Reverse Transcription kit (PrimerDesign, Southampton, UK) according to the manufacturer's instructions. Standardized quantities of RNA were used (500 ng total) for each biofilm condition to allow calculation of relative expression and comparisons between conditions.

### qPCR analysis of *C. albicans* virulence genes

Real‐time qPCR was performed as described previously (Cavalcanti *et al*. 2015; Morse *et al*. 2018). Putative virulence genes of *C. albicans* included adhesins involved in adhesion to biotic and abiotic surfaces and hyphal formation/promotion (agglutinin‐like sequence 3 (*ALS3*), epithelial adhesin 1 (*EPA1*), hyphal wall protein 1 (*HWP1*)); and production of secreted hydrolytic enzymes involved in nutrient acquisition, hyphal formation, tissue invasion and subsequent damage (phospholipase D1 (*PLD1*) and secreted aspartyl proteinases 4 and 6 (*SAP4*,* SAP6*)). The primer sequences were as detailed in Table S1. The analysis of relative gene expression was performed according to the ^ΔΔ^Ct method (Bustin *et al*. 2009) normalised to the *ACT1* housekeeping gene expression of *C. albicans*‐only biofilms. Statistical analysis of gene expression was performed using the ^ΔΔ^Ct values using one‐way analysis of variance, with Tukey's multiple comparisons test, at 95% confidence.

## Conflict of Interest

The authors declare no conflict of interest.

## Supporting information


**Table S1.** Forward (F) and reverse (R) primers used for evaluation of *Candida albicans* virulence gene expression by quantitative polymerase chain reaction (qPCR).
