# Supplementary material for: Modulation of Candida albicans virulence in in vitro biofilms by oral bacteria
Source: Lett Appl Microbiol. 2019 Mar 21;68(4):337–43. doi: 10.1111/lam.13145 (PMC6849710; doi:10.1111/lam.13145)
Supplement: Supplementary file 1 — Table S1. Forward (F) and reverse (R) primers used for evaluation of Candida albicans virulence gene expression by quantitative polymerase chain reaction (qPCR). [file LAM-68-337-s001.docx]

**Table S1.** Forward (F) and reverse (R) primers used for evaluation of *C. albicans* virulence gene expression by quantitative polymerase chain reaction (qPCR)

| Target gene | Sequence (5’ 🡪 3’) | Reference |
| --- | --- | --- |
| ACT1  Housekeeping Gene | F – TGCTGAACGTATGCAAAAGG  R – TGAACAATGGATGGACCAGA | (Alves *et al.* 2014; Cavalcanti *et al.* 2015; Morse *et al.* 2018) |
| ALS3  Agglutinin-Like Sequence 3 | F – CTGGACCACCAGGAAACACT  R – GGTGGAGCGGTGACAGTAGT | (Bandara *et al.* 2013; Alves *et al.* 2014; Cavalcanti *et al.* 2015; Morse *et al.* 2018) |
| HWP1  Hyphal Wall Protein 1 | F – TCTACTGCTCCAGCCACTGA  R – CCAGCAGGAATTGTTTCCAT | (Alves *et al.* 2014; Cavalcanti *et al.* 2015; Morse *et al.* 2018) |
| PLD1  Phospholipase D 1 | F - GCCAAGAGAGCAAGGGTTAGCA  R – CGGATTCGTCATCCATTTCTCC | (Alves *et al.* 2014; Cavalcanti *et al.* 2015; Morse *et al.* 2018) |
| SAP4  Secreted Aspartyl Proteinase 4 | F – GTCAATGTCAACGCTGGTGTCC  R - ATTCCGAAGCAGGAACGGTGTCC | (Alves *et al.* 2014; Cavalcanti *et al.* 2015; Morse *et al.* 2018) |
| SAP6  Secreted Aspartyl Proteinase 6 | F – AAAATGGCGTGGTGACAGAGGT  R - CGTTGGCTTGGAAACCAATACC | (Alves *et al.* 2014; Cavalcanti *et al.* 2015; Morse *et al.* 2018) |
